# Artificial Intelligence-Enhanced Flexible Sensors for Human Motion and Posture Sensing

**DOI:** 10.3390/s26051562

**Published:** 2026-03-02

**Authors:** Yiru Jiang, Tianyiyi He

**Affiliations:** 1 Artificial Intelligence Research Institute, Shenzhen MSU-BIT University, Shenzhen 518100, China; 3220241243@bit.edu.cn; 2 Guangdong-Hong Kong-Macao Joint Laboratory for Emotional Intelligence and Pervasive Computing, Shenzhen 518100, China

**Keywords:** AI-enhanced sensors, flexible sensors, self-powered sensors, human motion monitoring, intelligent wearable systems

## Abstract

In the era of Industry 4.0, artificial intelligence technology is experiencing rapid development, and the integration of artificial intelligence (AI) with flexible sensors has emerged as a transformative approach for human motion and posture sensing. This paper explores the advancements in AI-enhanced flexible sensors, focusing on the application of flexible sensors on various parts of the human body. Flexible sensors, due to their conformability and sensitivity, are ideal for capturing the dynamic and subtle movements of the human body. AI algorithms, particularly machine learning and deep learning techniques are employed to process the complex data streams from these sensors, enabling the accurate recognition and prediction of various human postures and motions. The combination of these technologies overcomes the limitations of traditional sensing systems, offering higher precision, adaptability, and real-time feedback. It can be applied to healthcare for rehabilitation monitoring, sports for performance enhancement, and human–computer interaction for intuitive control. This review also discusses the challenges such as sensor reliability, data privacy, and power management. The future outlook emphasizes more sophisticated AI models and deeper technology integration, promising a seamless integration into everyday life for enhanced human–machine interaction and health monitoring.

## 1. Introduction

Human body motion refers to the movement and posture changes in various parts of the human body in space. The analysis of human motion involves tracking, detecting, and recognizing the three-dimensional movements of subjects, offering valuable insights into physical activities and behaviors [1,2,3,4]. Human motion recognition demonstrates versatile applications across diverse domains, including anomaly detection systems, identity verification protocols, and enhanced security surveillance optimization. Within medical rehabilitation frameworks, motion analysis technology serves as a critical tool for facilitating patient rehabilitation training and continuous health monitoring, enabling the development of data-driven personalized treatment strategies [5,6,7,8]. Moreover, in virtual and augmented reality systems, accurate motion capture is essential for creating immersive and interactive environments, where the tight coupling between physical movements and virtual feedback substantially enhances spatial perception and embodiment experiences [9,10].

Human action recognition through computer vision technology operates by extracting spatiotemporal features and tracking kinematic coordinates from image/video sequences. Although it achieves high-precision detection capabilities, this methodology necessitates strict lighting controls and introduces privacy vulnerabilities, fundamentally restricting its operational scope in personal environments. Another limitation of image-based motion recognition is its inability to ensure continuous monitoring as the subject transitions between different locations, thereby restricting its applicability for consistent user-specific analysis [11,12]. Radar-based sensing represents another widely adopted modality, typically used in smart homes and medical applications. However, radar systems often struggle with real-time responsiveness to rapidly changing motions and exhibit limited robustness against environmental interference during daily activities. In contrast, wearable sensor-based approaches have gained increasing attention due to their ability to provide continuous, user-specific, and context-aware motion monitoring. For example, Inertial sensors, incorporating accelerometers and gyroscopes, enable continuous monitoring and precise recording of human kinematic states during biomechanical analysis [13,14,15,16]. Similarly, environmental sensors provide insights into the interaction between the human body and its surroundings. For instance, Avellar et al. developed a polymer optical fiber-based smart carpet integrating 20 sensing points with 20 cm spatial spacing to quantify ground reaction forces in the range of 10–50 N and extract spatiotemporal gait parameters, achieving a mean spatial error of ~2.9% during walking tests. Such floor-integrated systems enable distributed force mapping and gait event detection over meter-scale areas, yet remain limited in portability and user-specific adaptability [17].

Despite these advances, conventional wearable sensors are predominantly rigid, which compromises wearing comfort, conformability, and long-term usability, particularly under dynamic or prolonged usage conditions [18,19,20]. To address these limitations, flexible sensors have emerged as a promising alternative. Flexible sensors are typically made of flexible materials such as polyimide (PI) [21,22], polyester (PET) [23,24], and polydimethylsiloxane (PDMS) [25], making them flexible, bendable, foldable, and even stretchable. When laminated onto the skin or integrated into textiles, these sensors can capture subtle deformations (strains as low as 0.1%) and large-range motions (strains up to 60–100%) with a fast response time (typically <50 ms) and high signal fidelity. They can be applied to various body parts, such as the eyes [26,27,28], throat [29,30,31], joints [32,33], and feet [34,35].

The transduction performance of flexible wearable devices is fundamentally governed by their sensing mechanisms, which convert mechanical stimuli from human motion into measurable electrical signals. Primarily, these include piezoresistive sensing [36], which relies on changes in electrical resistance during deformation; capacitive mechanisms [37], which detect variations in electrode geometry or dielectric properties; and piezoelectric effects [38,39,40], where electric charges are generated in response to mechanical stress. Additionally, triboelectric nanogenerators leverage contact electrification and electrostatic induction to enable self-powered sensing [41]. These fundamental mechanisms determine the signal characteristics and data quality, forming the essential physical foundation for subsequent signal acquisition.

In the era of Industry 4.0 [42], artificial intelligence (AI) has become a transformative force across scientific and engineering disciplines. Originating from early concepts such as artificial neurons and the Turing test, AI has undergone cycles of rapid development, stagnation, and resurgence, culminating in explosive growth in the 21st century. Machine learning is a crucial branch of AI, focusing on enabling computers to learn from data patterns and make inferences or predictions [43,44]. In particular, deep learning methods based on artificial neural networks can automatically extract hierarchical feature representations from complex and high-dimensional data, representing a paradigm shift in data-driven modeling [45]. When integrated with sensing technologies, AI offers powerful tools to overcome intrinsic challenges such as signal nonlinearity, sensor drift, inter-subject variability, and multimodal data fusion. Consequently, AI-enhanced sensor systems have demonstrated remarkable potential in applications including early disease detection [46], personalized treatment [47], motion performance analysis [48], emotion recognition [49], and more.

**Figure 1:**
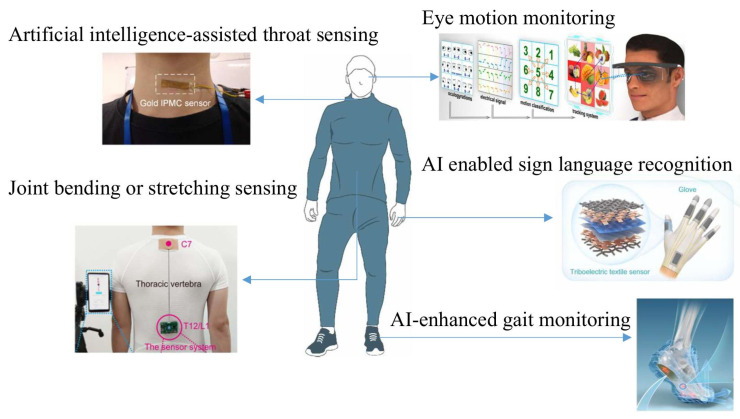
A brief overview of AI-enhanced body motion and posture detection [31,33,50,51,52].

In this review, we primarily discuss the research progress, applications, and future development directions of AI-enhanced flexible wearable sensors for human motion and posture sensing (Figure 1). We first introduce representative approaches for human motion detection, including vision-based, radar-based, and wearable sensor-based methods, followed by a discussion of the limitations of traditional rigid sensors and the advantages of flexible sensing technologies. We then examine how artificial intelligence facilitates advanced signal processing, feature extraction, and intelligent interpretation of flexible sensor data for diversified motion monitoring. Specifically, Section 2 and Section 3 summarize the characteristics of different body parts and review representative applications of flexible wearable sensors for subtle and large-range motion detection, respectively. Section 4 focuses on integrated AI–sensor systems for comprehensive human kinematic monitoring, including hand and foot motion analysis. Finally, we discuss current challenges and outline future perspectives on the convergence of flexible sensing, artificial intelligence, and intelligent systems, highlighting their potential to enhance convenience, safety, and intelligence in everyday life.

## 2. Flexible Sensors for Subtle Motion Detection

Flexible sensors are typically fabricated from materials possessing high bendability and flexibility. This allows them to conform seamlessly to non-planar surfaces, such as human skin. These sensors can detect subtle pressure changes, with their sensitivity often quantified by resultant changes in electrical signals. To bolster sensitivity, researchers employ microstructures and highly elastic or soft materials. As a result, flexible sensors are particularly effective at monitoring small-scale movements, such as eye and throat motions, where high precision is essential.

### 2.1. Eye Motion

Eye movement analysis serves as a vital method for investigating the human visual system and cognitive functioning. Within this realm, two critical components emerge: blinking movements and eye rotational movements. Flexible sensors have exhibited considerable potential in detecting eye movements, primarily due to their superior comfort and ability to conform to the shape of the eye [53].

Blinking serves as the eye’s natural protective mechanism and offers insights into emotional and cognitive states [54]. Flexible sensors can capture skin electrical signal changes generated by blinking, as this process involves periocular muscle activity producing detectable potential differences. Eye movement, comprising gaze, saccade, and smooth pursuit, is the primary mode of visual attention transfer. Flexible sensors enable eye movement monitoring by detecting subtle changes in the periorbital skin. The high sensitivity and rapid response time of flexible sensors facilitate accurate tracking of quick eye movements [55].

Triboelectric nanogenerators (TENGs) are emerging energy-harvesting devices that convert mechanical stimuli into electrical signals through the combined effects of triboelectrification and electrostatic induction. Their applications span self-powered sensing, wearable electronics, the Internet of Things (IoT), and even large-scale energy harvesting. A self-powered eye-movement triboelectric nanogenerator (TENG) sensor was developed to wirelessly detect both voluntary and involuntary eyelid motions [56]. The study introduces a non-attached electrode–dielectric triboelectric sensor (NEDTS), in which conductive electrodes are separated from dielectric layers by approximately 1.5 cm. The sensor delivers peak-to-peak voltages of 300–600 mV during involuntary blinks, while voluntary blinks exhibit significantly higher amplitudes of ~1.5 V compared with ~0.9 V for involuntary events, enabling reliable discrimination between the two modes. The system achieves a signal-to-noise ratio of ~13 dB and a response speed sufficient to resolve fast blinks (~200 ms) from slow blinks (>500 ms), supporting fatigue-monitoring applications. Leveraging this triboelectric–electrostatic human–machine interface, users with disabilities can perform hands-free operations, including cursor manipulation, remote vehicle control, and drone navigation, through predefined blink pattern.

**Figure 2:**
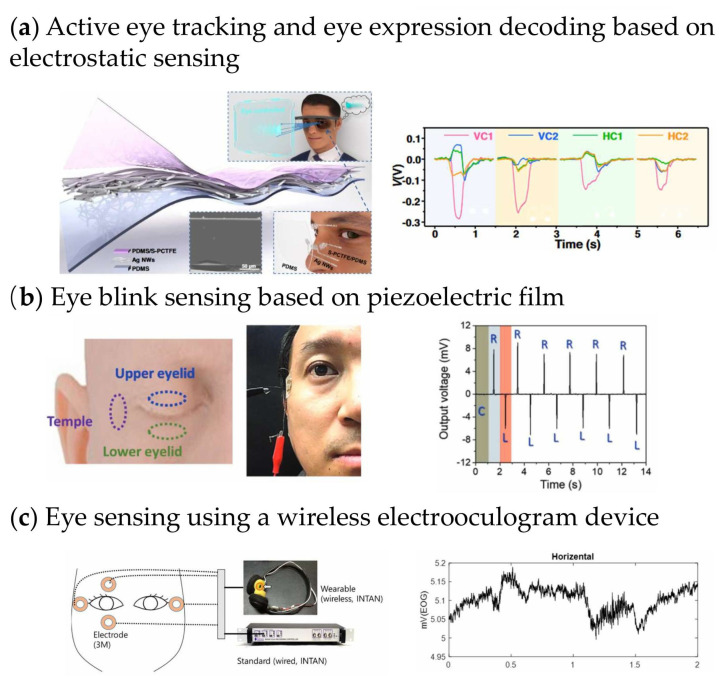
Flexible sensors for eye motion monitoring. (**a**) An active eye-tracking system; electrostatic signals of the interface responding to eye movements including supraversion, infraversion, laevoversion and dextroversion [50]. (**b**) Highly sensitive skin-attachable eye-movement sensor using flexible nonhazardous piezoelectric thin film; output voltage of eye movement [57]. (**c**) A wireless electrooculogram wearable device using a new type of conductive polymer fiber electrode; the Electrooculography (EOG) pattern of “horizontal” eye movement from one channel EOG system [58].

Active eye tracking technology involves the real-time monitoring and analysis of eye movements and gaze direction using active sensors or controlled stimuli. This technology plays a crucial role in various applications, such as human–computer interaction, virtual reality, and medical diagnostics. Figure 2a [50] shows a transparent, flexible, and ultra-persistent electrostatic sensing interface for realizing an active eye-tracking system based on the electrostatic induction effect. The electrostatic sensing interface is designed as a three-layer structure consisting of a pre-charged composite dielectric double layer and a retractable rear electrode Ag nanowire, which is adhered to the polydimethylsiloxane substrate. The optimized interface achieves an exceptional electrostatic charge density of 1671.10 μC·m^−2^ with an ultra-high charge-keeping rate of 96.91% after 1000 non-contact operation cycles, and maintains stable output performance at humidity levels below 90% and air flow speeds up to 8.1 m·s^−1^. The triple-layer structure, combined with the rough-surface Ag NW electrode that enhances inherent capacitance from 426.71 nF to 472.63 nF, enables amplified charge storage effect and long-term stability. This allows the active eye-tracking system to decode oculogyria-induced eyelid movements and related skin fluctuations, ultimately enabling detection of eye movements with an angular resolution of 5° and response time within 1.002 ms for real-time human–computer interaction.

Based on the piezoelectric effect, piezoelectric sensors can detect minute variations in force or pressure without requiring an external power supply, enabling long-term stable operation. Earlier studies placed piezoelectric sensors based on lead zirconate titanate (PZT) or zinc oxide (ZnO) directly on the upper eyelid to monitor eye movements; however, these approaches suffered from limited wearing comfort and potential safety concerns associated with lead toxicity, or insufficient sensitivity that necessitated additional signal amplification. To address these limitations, Kim et al. developed a highly sensitive, skin-attachable eye-movement sensor positioned near the temple, enabling non-invasive, comfortable, and accurate signal acquisition [57]. The device is fabricated from single-crystalline III-N (GaN) thin films using a layer-transfer process, providing excellent mechanical flexibility and durability without performance degradation. The flexible piezoelectric eye-movement sensor (F-PEMS) exhibits high sensitivity during eyelid closure, generating output voltages of approximately 97.9 mV at the upper eyelid, 78.3 mV at the lower eyelid, and 32.3 mV at the temple, accompanied by signal-to-noise ratios exceeding 40 dB, specifically reaching 49.3 dB, 47.9 dB, and 40.1 dB, respectively, confirming its suitability for wireless data transmission. The sensor shows a rapid response, enabling clear resolution of the four characteristic stages of a normal blink, including closing, closure, opening, and gazing, and supports detection of rapid eye movements at frequencies up to 1.9 Hz. As illustrated in Figure 2b, lateral eye motion from center to right and then to left produces positive voltages of approximately 10 mV when approaching the sensor and negative voltages when moving away. This platform enables quantitative extraction of key ocular metrics, including blink rate, blink duration, and the proportion of eyelid closure (PERCLOS), which are essential indicators for fatigue and drowsiness assessment. In addition, the capability to track lateral eyeball movements provides potential diagnostic value for neurological conditions such as Attention-Deficit/Hyperactivity Disorder (ADHD), stroke, autism, Alzheimer’s disease, and Parkinson’s disease, and also supports emerging applications in Virtual Reality/Augmented Reality (VR/AR) systems.

Electrooculography (EOG) measures eye movement by recording the potential difference between the cornea and retina. However, conventional EOG systems are poorly suited for daily-life monitoring due to electrode discomfort and wired constraints. Moon et al. [58] reported a wireless wearable EOG interface using semi-dry conductive polymer fiber electrodes fabricated by electrospinning, together with a compact Bluetooth Low Energy wearable module shown in Figure 2c. The graphene-infused polylactic acid electrodes combined with electrolytic gel achieved a low skin–electrode impedance of approximately 37 kΩ at 50 Hz, representing a substantial reduction compared with both dry and conventional wet electrodes, while maintaining excellent stability with negligible impedance drift over 15 min. The system supports high-rate signal acquisition and up to 8 h of continuous wireless operation. Two eye-motion indices, Eye Beat Rate and Eye Beat Rate Variability, were introduced to enable real-time classification of rest, blink, and horizontal movement states. Owing to its miniaturized form factor, the device can be integrated into a headphone-like wearable, offering improved concealment and comfort relative to electronic tattoos. Moreover, the semi-dry electrode design eliminates the need for skin preparation and frequent replacement, making the platform well suited for continuous daily monitoring applications such as drowsiness detection in assisted driving.

### 2.2. Throat Motion

While the detection of eye movements demonstrates the potential of flexible sensors in monitoring subtle and localized motions, their applicability extends to other fine movements, such as those of the throat. The application of flexible sensors in throat motion detection is mainly reflected in medical health monitoring and human–computer interaction technology. First, the flexible sensor can detect the movement of the throat, which is of great significance for the analysis of vocal cord vibration and the diagnosis of speech pathology. Second, flexible sensors are able to pick up tiny changes in vocal cord vibrations that contain a wealth of language information. Using these differences in signals, speech recognition can be performed. Third, the flexible sensor can also monitor respiratory movement, which is very important for the diagnosis and monitoring of diseases such as sleep apnea hypopnea syndrome [59]. Liu et al. integrated a flexible piezoelectric nanogenerator with an elastic band that can monitor human breathing in real time [60]. By integrating flexible piezoelectric nanogenerators with elastic straps, human breathing conditions can be monitored in real time. Compared with traditional invasive monitoring devices, flexible sensors provide a non-invasive way of monitoring, reducing patient discomfort and the risk of infection, and increasing patients’ willingness to be monitored.

Biometric signal recognition is central to health monitoring applications. A hierarchically resistive skin sensor was developed using three stacked conductive layers embedded in PDMS, forming a multimodal platform capable of responding to different strain ranges [29]. This architecture produces a staircase-like resistive response, in which small strains below 3% are dominated by the top layer with a gauge factor exceeding 10^2^, medium strains from 3% to 35% are governed by the middle layer with a gauge factor of 2111, and large strains up to 50% are captured by the bottom layer. Unlike conventional single-mode sensors, this design enables simultaneous detection of multiple throat-related activities from a single resistance signal, including speech, heartbeat, breathing, touch, and neck motion. High sensitivities are achieved for both acoustic vibration at 200 Hz and pressure in the 10–100 kPa range. To decouple these mixed physiological signals, a frequency–amplitude-based neural network was introduced, allowing automatic separation of multiple biometrics. The system successfully classifies 11 combined activities, including speech commands, neck movements, and touch, with an accuracy of 92.73 ± 0.82%, while concurrently extracting respiration and heart rate.

**Figure 3:**
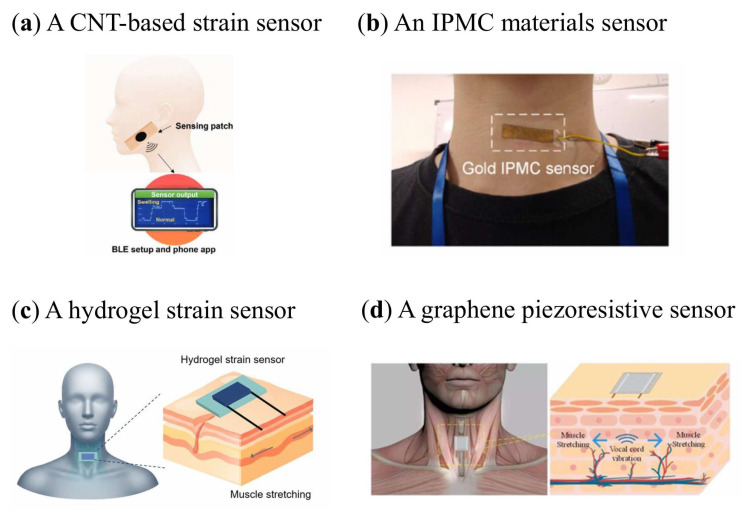
Flexible sensors for throat motion sensing. (**a**) A robust wearable Point-of-Care CNT-Based strain sensor [30]. (**b**) A smart and non-invasive throat sensor based on ionic polymer–metal composite (IPMC) [31]. (**c**) A hydrogel strain sensor [61]. (**d**) A flexible graphene piezoresistive sensor for speech recognition [62].

Figure 3a focuses on Point-of-Care (POC) detection, developing a highly flexible and stretchable piezoresistance-sensing patch that is capable of recording muscle expansion or relaxation in real time and can therefore serve as a next-generation POC sensor [30]. The sensor demonstrates excellent electromechanical performance with a high gauge factor of 14.5 for in-plane stretching up to 20% strain, exhibiting low hysteresis and outstanding repeatability over 50 cycles. Notably, the sensor can respond to small-to-large-scale strains (higher than 100%), with a minimum strain resolution of 0.033. Furthermore, the sensor integrates a Bluetooth Low Energy (BLE) system operating at a sampling rate of 8 Hz to track muscle activity in real time and wirelessly transmit the output signal to a smartphone app, enabling convenient remote monitoring. This work provides a new wearable sensing solution for real-time monitoring of throat-related diseases such as parotid gland swelling caused by mumps or flu, with significant potential applications in personalized healthcare systems.

An AI-assisted throat sensor based on IPMC represents a new class of non-invasive, self-powered, and flexible medical devices (Figure 3b) [31]. The sensor detects throat muscle activity through self-generated electrical signals arising from cation migration within the IPMC matrix. With gold electrode integration, the device exhibits a linear pressure response over a range of 40–160 kPa with R^2^ approaching 0.97 and a sensitivity of 1.5 × 10^−6^ mV/kPa, generating sub-millivolt output voltages that are approximately 1.5 times higher than those of silver-coated counterparts, highlighting the importance of electrode stability. Using a support vector machine classifier, the system achieves around 95% accuracy in recognizing throat motion patterns. Beyond basic activity detection, including swallowing, humming, nodding, and coughing, the platform can further distinguish dynamic actions based on waveform characteristics such as amplitude and response speed, demonstrating its potential for intelligent perception in complex physiological environments.

Hybrid Mode speech recognition and interaction technology using wearable artificial throat is an innovative voice interaction solution that combines the perception of acoustic signals and mechanical motion to improve speech recognition accuracy and interaction in noisy environments. At the heart of the technology lies a smart, graphene-based wearable artificial throat (AT) that is highly sensitive to human speech and phonation-related movements. The AT exhibits linear sensitivity to relative strain below 1% and maintains uniform response over 1000 bending fatigue tests, demonstrating excellent mechanical stability. Its frequency response covers the human voice range up to 2000 Hz with particular sensitivity to audio signals, and the device can generate sound pressure levels of nearly 60 dB through the thermoacoustic effect at a safe operating voltage of 5 V. The artificial throat can detect low fundamental frequency signals while maintaining noise resistance, allowing it to maintain recognition in ambient noise above 60 dB [63]. The research team also used artificial intelligence models for speech recognition and synthesis of signals perceived by the artificial throat, achieving high-precision recognition of basic speech elements (phonemes, tones, and words). The experimental results show that the mixed mode speech signals collected by artificial throat can recognize basic speech elements with an average accuracy of 99.05%.

Another example is the development of a stretchable hydrogel strain sensor with an ultra-low detection limit and excellent anti-jamming capabilities, designed for smart throat speech recognition, as shown in Figure 3c [61]. The hydrogel strain sensor has an ultra-low strain detection limit (0.1%), a good strain coefficient (gauge factor, GF = 8.29), and high tensile property (600%). This allows the sensor to continuously monitor the full range of human movements as a wearable device and to detect subtle throat voice movements as a voice interface. Further classified by a transfer learning algorithm based on the ResNet50 neural network, the sensor can identify ten common words that express the needs of the body with 100% accuracy. This technology opens up the possibility for patients or infirm patients with voice disorders in hospitals to express their needs and provides new insights into the applications of next-generation smart wearables in healthcare, medical treatment, diagnosis, treatment and rehabilitation.

Laryngeal speech signal detection based on flexible piezoresistive sensors addresses the challenge of environmental noise interference in traditional speech acquisition and recognition. Professor Kai’s team at Yanshan University has proposed a flexible graphene sensor to detect human acoustic vibration signals as shown in Figure 3d [62]. The sensor is prepared using chemical vapor deposition (CVD) and imprinting technology, and has a cylindrical micro-surface structure substrate, which not only improves the conformal coating capability of the sensor, but also greatly improves its sensitivity. The sensor has an average voltage gain of about 48 dB over a frequency range of 200–2500 Hz, which basically covers human speech frequencies. The researchers also conducted bilingual tests in both Chinese and English, and the results showed that the graphene speech sensor is sensitive enough to extract the characteristics of sound waves for speech recognition.

## 3. Flexible Sensors for Large-Range Motion Detection

With increasing societal focus on health and sports performance, real-time monitoring of human movement has emerged as a prominent research area [64]. Wearable flexible sensors have demonstrated significant potential in monitoring human motion parameters, such as joint angles, velocity, and acceleration. These sensors provide valuable data for sports training and health monitoring applications. Concurrently, in the domain of medical rehabilitation, accurate recognition of patient movements is vital for effective rehabilitation training. Flexible sensors enable continuous dynamic monitoring, assisting medical professionals and rehabilitation specialists in assessing the progress and effectiveness of a patient’s recovery. Extensive scientific research has been conducted on wearable flexible sensors for monitoring various body movements [65,66,67]. This section presents a comprehensive review of wearable flexible sensors, categorized into three primary aspects: hand motion monitoring, joint motion monitoring, and gait monitoring.

### 3.1. Hand Gesture Recognition

Gestures are one of the most natural forms of human communication. In comparison with traditional input devices like keyboards and mice, gesture recognition technology facilitates a more intuitive mode of interaction with devices. Furthermore, gesture recognition can provide immersive interactive experiences for VR and AR applications [68,69]. The development of flexible sensors offers the possibility of directly measuring finger movement behavior; accurate and cost-effective gesture recognition can be achieved by placing the sensor on the finger or integrating it into a data glove. The research of flexible sensors involves a variety of materials and structural designs, graphene composites [70], conductive polymers [71] and nanomaterials are used to improve the electrical and mechanical properties of sensors. In addition, there are some new textile materials [72] have been discovered.

A wearable strain sensor based on ultra-flexible polyurethane (PU) yarns with a PDMS-permeated multilayer sheath has been developed for smart textile applications [73]. This sensor is designed to meet the needs of smart textiles, wearables and biomedical electronics for water resistance, high specification factor and excellent stability. Specifically, the sensor exhibits an ultra-high sensitivity with a gauge factor (GF) of up to 661.59. It possesses a broad strain-sensing range of up to 75% and maintains outstanding stability and repeatability over 10,000 stretching–releasing cycles. In addition, this sensor can be easily integrated into textiles, such as medical textile bandages and textile gloves, and has an important role in monitoring finger-bending movement. The smart glove is capable of comprehensively detecting human hand movements and enables the manipulation of the robotic hand to perform actions such as movement, capturing, holding, and grasping of various objects.

**Figure 4:**
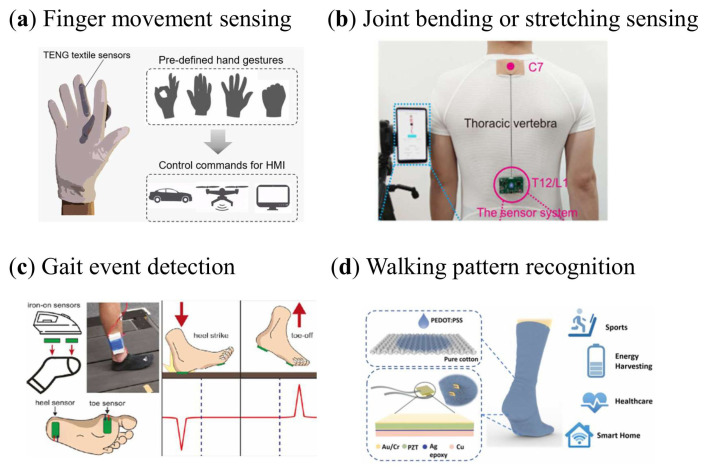
(**a**) A glove-based sensor with a novel minimalist design concept [75]. (**b**) A retractable and wearable badge-like sensor [33]. (**c**) Smart socks with Flexible ironing sensors; the red line indicates the expected output result of the corresponding action [34]. (**d**) Self-powered and self-functional cotton socks [35].

TENG’s invention is considered a landmark discovery in the field of mechanical energy generation and self-drive systems, providing an entirely new model for the efficient collection of mechanical energy [74]. As shown in Figure 4a, based on TENG, researchers utilized conductive polymer poly(3,4-ethylenedioxythiophene):poly(styrenesulfonate) (PEDOT:PSS) coated textiles and silicone rubber coated on the outer surface of gloves to integrate into cotton gloves through a simple design, providing a new intuitive and self-powered control technology [75]. The design employs an innovative and highly simplified approach, streamlining the device structure and signal processing method, thereby reducing manufacturing costs and facilitating large-scale production. The textile-based TENG sensors deliver an open-circuit voltage of 0.6–0.8 V, with linear responses to bending angles and movement speeds, and exhibit high durability, retaining most of their output after tens of thousands of cycles. Utilizing a glove interface, researchers achieved wireless control of a toy car’s movement on the ground at varying speeds and directions by adjusting the bending angles and contact forces. Additionally, this glove can be applied to gesture recognition tasks such as letter writing and cursor control, offering an intuitive and user-friendly operational experience.

### 3.2. Joint Rotation Detection

The skin surface of joint areas is inherently uneven. Flexible sensors, owing to their comfortable and stretchable characteristics, offer a significant advantage in detecting joint movements. The sensors can effectively monitor joint flexion, extension, and rotation, providing valuable data for motion analysis and rehabilitation training.

The flexible, stretchy, breathable, and sweatproof all-nanofiber ionic electronic tactile sensor provides a new approach for continuous and comfortable knee motion monitoring [32]. Based on an electrospun thermoplastic polyurethane (TPU) nanofiber platform, the sensor features a multi-layer, interwoven nanofiber network containing numerous micropores and channels that give the sensor superior air permeability, sweat resistance, heat dissipation and biocompatibility, ensuring long-term wear comfort. Specifically, the all-nanofiber iontronic tactile sensor exhibits outstanding sensing performance, featuring a high sensitivity of 8.14 kPa^−1^ in the low-pressure range (less than 22.4 kPa), a broad pressure detection range of 0 to 300 kPa, and excellent stability characterized by maintaining consistent electrochemical performance after 1000 continuous compression cycles without electrolyte leakage. By mounting three separate sensor units at the knee joint edge, as well as a 5 × 5 sensor array firmly attached to the entire knee joint area, single point and spatial pressure distribution can be continuously and non-invasively monitored. The accumulated sensory data revealed the real load distribution of the knee joint during long walking and jogging, reflecting the health status of the knee joint in real time.

A retractable and wearable badge-like sensor, the raster structure-based friction triboelectric nanogenerator, has been developed for real-time monitoring of the bending or extension of the human joints and spine [33]. The sensor shown in Figure 4b, which is inspired by the badge scroll, is capable of simultaneous extension and contraction with human movement, with high sensitivity (8 V/mm), minimum resolution (0.6 mm), excellent durability (more than 120,000 extension cycles) and low lag. The researchers used this sensor to record joint movements such as knee/arm bending and neck/waist twisting, demonstrating real-time monitoring capabilities. In addition, by anchoring the sensor along the spine, it detects changes in the shape of the spine, demonstrating its potential application in everyday spinal monitoring.

### 3.3. Gait Monitoring

Gait Monitoring refers to the real-time or regular recording and analysis of the movement characteristics and parameters of the human body during walking through various sensors and technical means. The core of gait monitoring lies in capturing the unique biomechanical and kinematic parameters of an individual’s gait, which encompass various characteristics such as the gait cycle, stance and swing phases, body posture, and walking velocity [76,77,78]. It has a wide range of applications in security monitoring, health monitoring and auxiliary medicine. Piezoresistive sensors are the most commonly used sensors for gait recognition and can be made from a variety of materials, such as nanocomposites, graphene, etc.

Flexible ironing sensors embedded in smart socks for gait event detection is an innovative wearable technology that leverages the benefits of flexible electronics and smart textiles to provide a low-cost, washable and highly versatile solution for gait analysis. As shown in Figure 4c, this study uses a washable ironing textile pressure sensor to collect biometric data [34]. The researchers integrated a washable ironing device into the sock and compared its performance against gold-standard force-platform data. This embedded sensor assesses its efficacy by detecting gait events, including heel strikes and toe-off movements. The iron-on textile pressure sensor adopts an ultrathin multilayer architecture with an overall thickness below 0.8 mm, exhibiting sensitivities of 2.19 mV kPa^−1^ at the heel and 4.31 mV kPa^−1^ at the toe. It maintains a linear response over a pressure range of 65–450 kPa and delivers rapid response times of approximately 4 ms for heel sensing and below 2 ms for toe sensing, enabling real-time capture of gait events. Biomechanical evaluations demonstrated that the sensor achieved superior performance in gait event detection, accurately identifying over 96 percent of heel strikes and toe-off events. This sensor can not only detect gait events, but also provide detailed information about gait patterns, which is expected to be widely used in the field of sports science and smart home.

Self-powered and self-functional cotton socks named S2-sock (Figure 4d) using a hybrid piezoelectric and triboelectric mechanism are an innovative wearable device that combines the benefits of piezoelectric materials and TENG for healthcare and motion monitoring [35]. The socks combine energy harvesting and physiological signal sensing by integrating a fabric-based TENG coated with PEDOT:PSS and a lead zirconate titanate (PZT) piezoelectric chip. The sock can generate an output power of 1.71 mW under a light jump frequency of 2 Hz and a load resistance of 59.7 MΩ. The research team further investigated the impact of varying ambient humidity, temperature, and weight on the S2-sock, thereby validating its multifaceted capabilities such as energy harvesting, walking pattern recognition, motion tracking, gait sensing, and sweat sensing. These functionalities have potential applications in smart homes, healthcare, and exercise monitoring.

## 4. AI-Enhanced Systems for Human Kinematic Monitoring

Flexible wearable sensors enable continuous, high-resolution acquisition of kinematic signals associated with human motion and posture, including joint deformation, muscle activity, pressure distribution, and spatiotemporal movement patterns. However, data generated by these systems are inherently high-dimensional, multimodal, and often exhibit strong nonlinearity, hysteresis, and inter-subject variability, making reliable interpretation challenging when relying solely on conventional signal processing and rule-based methods. To address these limitations, machine learning (ML) techniques that are capable of automatically learning discriminative representations from raw sensor data have been increasingly integrated into wearable sensing systems (Table 1). This integration allows complex motion patterns to be accurately identified, classified, or regressed, thereby enabling applications such as robust motion tracking, posture recognition, and movement quality assessment.

A central challenge in flexible wearable sensor-based motion and posture recognition is the limited scale of available datasets. When dealing with small-scale wearable datasets, the optimal methodology depends critically on the data characteristics and the target application. If the dataset is well-defined and the deployment distribution is expected to remain consistent with the training distribution, the primary objective is effective model fitting. In such cases, traditional machine learning methods (e.g., Support Vector Machine, SVM [91], eXtreme Gradient Boosting, XGBoost [92]) are typically sufficient for simple data structures, while lightweight deep learning models are preferred for capturing more complex patterns [93]. When related datasets or pretrained models are available, transfer learning can further improve data efficiency by leveraging previously learned representations, often enhancing generalization under limited labeled data. However, “limited data” more commonly refers to scenarios where the training data is scarce, but the model must generalize to diverse and unseen test environments, leading to a distribution shift. In these situations, standard overfitting mitigation techniques like dropout or regularization may be inadequate. Under such conditions, data augmentation and synthetic data generation can serve as complementary strategies, introducing additional variability to better approximate the potential test distribution [94,95]. Furthermore, while self-supervised learning (SSL) has gained prominence for addressing label scarcity, it fundamentally requires a substantial volume of unlabeled data for pre-text tasks to learn robust representations before fine-tuning on small labeled subsets [96,97]. Consequently, in extreme scenarios where even raw data is scarce, carefully designed task-specific augmentation often remains practical baseline approach.

Beyond the selection of specific algorithms, the practical performance and responsiveness of these wearable systems are increasingly dictated by their computational deployment strategy. Cloud AI leverages powerful remote servers to process large-scale datasets and execute complex models, offering immense computational capacity but at the cost of network latency and potential privacy concerns. In contrast, Edge AI enables on-device data processing, which significantly reduces response time and enhances data security by minimizing external transmissions [98,99]. This local processing is particularly critical for real-time applications such as fall detection and instantaneous gesture control. The choice between these architectures depends on the specific trade-offs between computational complexity, power consumption, and the need for immediate feedback in practical wearable sensing scenarios [100,101].

To date, ML-assisted flexible wearable sensors have shown remarkable capability in capturing complex human motion, from fine hand gestures to full-body postures. Collectively, these developments highlight a clear transition from proof-of-concept demonstrations toward intelligent and scalable flexible wearable sensing systems, marking a critical step toward real-world human–machine interfaces and personalized health monitoring.

### 4.1. AI-Enhanced Hand Gesture Recognition

Advanced machine learning algorithms, such as deep learning models, support vector machines (SVM), and linear discriminant analysis (LDA), have been extensively employed to enhance the accuracy and real-time performance of hand gesture recognition systems [102]. The tight integration of ML with flexible wearable sensing is increasingly critical for decoding the complex, nonlinear signals arising from fine-grained finger motions [103]. However, achieving robust signal stability during intense physical activity remains a major challenge, as motion-induced noise and physiological signals often exhibit substantial spectral overlap, leading to pronounced signal distortion. To mitigate this issue, Chen et al. developed a noise-tolerant flexible wearable human–machine interface that integrates a six-channel Inertial Measurement Unit (IMU) and surface Electromyography (sEMG) modules within a multilayered architecture featuring a total thickness of ~2 mm and stretchability exceeding 20% [79]. To suppress complex motion artefacts, the system employs a LeNet-5-based convolutional neural network operating at a sampling rate of 100 Hz, combined with a sliding-window strategy using a 1 s window length and a 0.25 s stride. This design enables high-precision recognition of 19 gestures, achieving accuracies above 92% under diverse real-world conditions, including running, high-frequency vibrations, and underwater environments. Collectively, such approaches highlight the growing importance of tightly coupled hardware–algorithm co-design for maintaining sensing robustness in dynamic settings, particularly as IoT and 5G infrastructures continue to expand and demand reliable, real-time human–machine interaction.

A flexible wearable sensor array based on triboelectric technology was proposed to meet these requirements [104]. By leveraging triboelectrification and electrostatic induction between Ecoflex and the skin, the array enables sensitive detection of micro-displacements from finger tendons without requiring an external power supply. The Ecoflex-based sensors exhibit high durability over more than 10,000 loading cycles while maintaining stable voltage outputs. To improve signal quality, the raw data are processed using a Butterworth low-pass filter with a cutoff frequency of 50 Hz to suppress high-frequency noise. Based on six key features, including peak-to-valley intervals and zero-crossing points, nine hand gestures are classified using an LDA model implemented in the Scikit-Learn framework, achieving a recognition accuracy of 96.69% with low computational overhead. However, this performance currently depends on per-user calibration, as the classifier is trained on standard gesture templates to accommodate individual differences in signal amplitude and skin contact conditions. Overall, this work illustrates the potential of integrating self-powered sensing with lightweight machine learning for personalized human–machine interaction, while also highlighting the continued need for subject-specific model adaptation.

**Figure 5:**
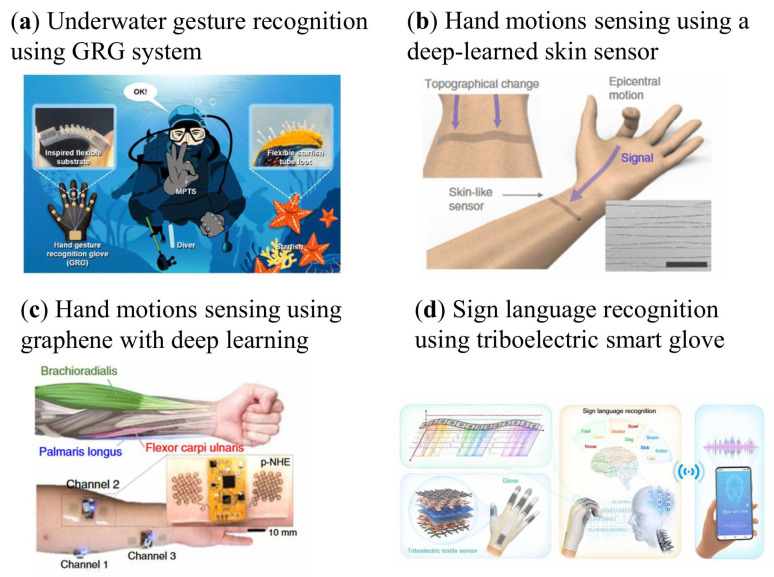
AI-enhanced flexible sensors for hand gesture sensing. (**a**) A glove with a built-in high-performance microcolumn tactile sensor [105]. (**b**) A deep learning-assisted sensor system [106] (**c**) Wireless flexible circuits with the developed functionalized conductive graphene material [107]. (**d**) The AI-assisted sign language recognition and virtual reality (VR) space two-way communication system [51].

Beyond terrestrial applications, AI-enhanced gesture recognition systems have been adapted for challenging underwater scenarios. As shown in Figure 5a, a wearable glove integrating high-performance microcolumn tactile sensors (MPTSs) was developed for marine communication [105]. Inspired by the tube feet of starfish, the MPTSs utilize a double-layer micropillar interlocking structure that achieves a superfast response/recovery time of ~23 ms and a wide sensing range from 5 Pa to 450 kPa, effectively handling the high-pressure gradients of underwater environments. This structural design ensures excellent reliability and repeatability across 10,000 cycles of repeated use. Crucially, the system incorporates a fully connected neural network (FCNN) for gesture classification. Through training on 76,800 data groups (80% training, 10% testing, 10% validation), the glove achieves accurate recognition of 16 gestures with 99.8% precision, facilitating real-time bidirectional communication for divers.

Deep learning techniques expand the capability of wearable systems by enabling automated feature extraction. As shown in Figure 5b, this skin-like sensing system utilizes highly sensitive, laser-induced crack structures to capture subtle wrist deformations [106]. To ensure real-time performance, the system operates at a 40 Hz sampling rate using a sliding window of 16 frames. This configuration allows the 5-layer LSTM network to extract spatiotemporal features for decoding five finger motions in real time. A key breakthrough is the Rapid Situation Learning (RSL) mechanism, which allows the system to adapt to new conditions (e.g., sensor displacement) without tedious long-term recalibration. RSL requires only 8 s of data collection per finger and approximately 5 min of retraining, significantly enhancing robustness and user readiness for applications in soft robotics and human–machine interaction.

Advances in nanomaterials and additive nanomanufacturing have further facilitated the development of flexible, multifunctional wearable electronics for gesture recognition. However, conventional fabrication techniques often rely on complex, multi-step processes that require costly cleanroom facilities and are prone to manufacturing errors [108,109]. To overcome these limitations, a research team at the Georgia Institute of Technology has developed an additive nanomanufacturing strategy for functional materials, enabling the realization of wireless, multilayer, seamlessly interconnected flexible hybrid electronic systems [107]. As shown in Figure 5c, an all-printed nanomembrane system uses functionalized conductive graphene to achieve high-fidelity recording of muscle activity. To capture the rapid bursts of EMG signals, the system operates at a 250 Hz sampling rate with 0.512 s data windows, enabling real-time robotic hand control with minimal latency. The graphene-based sensors exhibit excellent oxidation resistance and biocompatibility, maintaining stable electrochemical impedance over long-term skin contact. While the CNN-based classification achieves over 99% accuracy for seven classes, the system requires a brief user-specific training session to map the precise muscular trigger points of the individual to the seven finger-motion classes (opening hand, closing hand, bending thumb, bending index, bending middle, bending ring, bending pinky), ensuring high-fidelity translation from bioelectrical signals to control commands

More recently, meta learning has emerged as a powerful paradigm in artificial intelligence, aiming to enable models to rapidly adapt to new tasks and users by learning how to learn from prior experiences [110]. To address the challenge of user- and task-dependent variability, a substrate-less nanomesh receptor was developed [111]. The sensor mimics human cutaneous receptors by transducing micro-scale skin stretches into resistance changes with high sensitivity. Instead of relying on time-consuming per-user calibration, the system employs a time-dependent contrastive learning-based meta-learning framework. This approach allows the model to extract universal feature representations from unlabeled motion data, enabling the system to achieve rapid adaptation to new users with more than 80% accuracy within merely 20 transfer training epochs—a stark contrast to the thousands of epochs required by traditional supervised learning methods. The nanomesh structure provides a high degree of transparency and breathability (>40 mm s^−1^), ensuring that the sensor’s mechanical presence does not interfere with natural hand kinematics during diverse tasks like virtual keyboard input (achieving 85% accuracy for nine-class numpad typing and 82.1% accuracy for object recognition) and object manipulation.

Despite substantial progress, most existing sign language recognition gloves remain limited to the identification of discrete, isolated gestures and are unable to recognize continuous sentences, thereby failing to meet the practical communication demands of sign language users [112]. To overcome the limitation of recognizing only isolated gestures, an AI-assisted sign language recognition system was proposed for continuous sentence communication (Figure 5d) [51]. The system employs a triboelectric smart glove that enables self-powered sensing of multi-degree-of-freedom hand movements. By implementing a data sliding window approach and a segmentation-assisted deep learning model, the system identifies word units within a continuous data stream, effectively managing the temporal transitions between signs. The textile-based sensors demonstrate high durability and a stable voltage output, providing a robust signal source for the CNN to decode 50 words and 20 sentences. Notably, the system facilitates a high degree of user readiness; it can recognize new/never-seen sentences created by recombining known words with an accuracy of 86.67%, significantly reducing the need for exhaustive sentence-level training for every user.

### 4.2. AI-Enhanced Limb and Foot Motion Detection

The integration of AI with flexible sensor-based motion sensing systems also plays a critical role in lower-limb motion monitoring [113]. By learning individualized movement characteristics and behavioral patterns, AI algorithms can adapt to inter-subject variability and facilitate personalized healthcare and rehabilitation strategies. Moreover, the ability of AI to process and analyze large-scale, high-dimensional datasets collected from flexible wearable sensors allows the extraction of clinically and biomechanically meaningful information, supporting data-driven insights in sports science, ergonomics, and occupational health.

**Figure 6:**
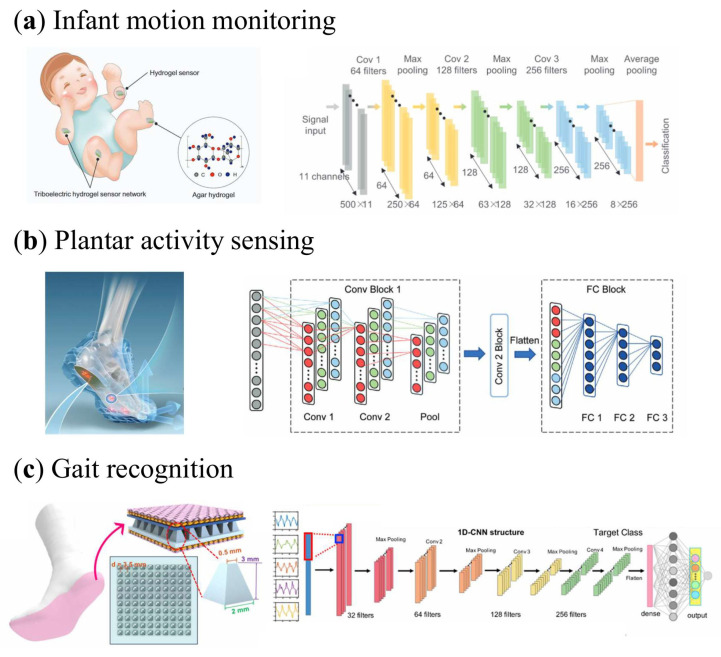
AI-enhanced Lower-Limb Motion and Fall Detection. (**a**) A deep learning assisted triboelectric hydrogel sensor network for infant care; architecture of deep learning algorithms [114]. (**b**) A self-powered insole based on triboelectric nanogenerators for plantar stress sensing; architecture of convolutional neural networks [52]. (**c**) A low-cost triboelectric smart sock; schematics of the process and parameters for constructing the 1D CNN structure [116].

Human movement serves as an important indicator of physiological and neurological status, and continuous motion monitoring enables early assessment of health conditions and environmental risks. This is particularly critical in infant care, where motor behavior often reflects developmental status and safety risks. As shown in Figure 6a, Guo et al. developed a smart infant care system utilizing a flexible, self-powered body area sensor network (BSN) composed of edible triboelectric hydrogel sensors [114]. The sensor network is characterized by a high signal-to-noise ratio (SNR) of 23.1 dB and a fast response time of 50 ms, enabling all-weather, low-latency monitoring of infant bumps and falls. These hydrogel sensors maintain high baseline stability over 3000 pressing–releasing cycles, ensuring long-term reliability for continuous monitoring. Assisted by a CNN algorithm that incorporates a 10% data calibration step, the system achieves a pattern recognition accuracy of 100%. This AI-integrated approach allows for real-time parsing of motor behaviors and quantitative estimation of collision risks, providing a robust and safe strategy for infant developmental monitoring and injury prevention.

In the field of lower-limb motion recognition, sensing technology is evolving from single-node sensors to high-density flexible arrays, necessitating AI models with superior spatiotemporal feature extraction capabilities. A representative study by Lian et al. exemplifies this trend by integrating a flexible sensor array with advanced deep learning for enhanced motion analysis [115]. The system utilizes a 4 × 4 sensor array to collect 16-channel muscle activity data via an ESP32-based circuit at a sampling rate of 120 Hz. To overcome the limitations of traditional 1D signal processing, raw data is converted into 80 × 80 grayscale relative position images (RPI) images that preserve spatial–temporal relationships. A novel deep learning architecture, MCRANet, is proposed, featuring multi-scale cascaded residual attention and cross-feature interaction modules to capture complex high-order feature relationships. The model was evaluated on 5720 samples across 10 motion categories performed by 10 participants. Experimental results demonstrate that the proposed framework achieves a remarkable recognition accuracy of 97.88%.

The perception of plantar stress is vital for sports science and medical diagnostics, offering deep insights into human biomechanics. As shown in Figure 6b, a self-powered sensing insole based on a TENG array was developed to map stress across four critical plantar positions (first metatarsal head, fifth metatarsal head, midfoot, and heel) [52]. A key technical advancement is its ability to decouple normal and shear stresses, achieving sensitivities of 7.95 mV/N and 10.15 mV/N, respectively, within the measurement range of 0–8 N and 0–30° angle. To ensure gait analysis, the system utilizes a 2.5 Hz sampling rate with 16-channel synchronous measurement. By integrating a CNN to decode spatiotemporal stress patterns from multi-channel signals, the system identifies abnormal gait with 97.03% average accuracy, highlighting its potential for disease diagnosis and rehabilitation. This demonstrates its significant promise for personalized athletic performance evaluation and clinical applications.

Gait analysis also provides rich sensory information related to personal identity, physical condition, and daily activity patterns. As shown in Figure 6c, a low-cost, self-powered smart sock utilizing frustum-patterned triboelectric nanogenerators has been developed [116]. The sensors exhibit a threefold higher sensitivity compared to flat-structured sensors (which saturate at 0.4 V kPa^−1^) and a wide sensing range of over 200 kPa, enabling precise capture of both subtle movements and high-impact gaits. To optimize the balance between feature density and recognition speed, the system uses a 4 s sliding window with 50% overlap, which effectively doubles the training data for the 1D CNN model. While the system achieves an accuracy of 96.67% for five general human activities, identifying individual users requires collecting training data from each participant to achieve 93.54% identification accuracy among 13 participants. These results highlight the smart sock’s potential for pervasive healthcare and identity verification in smart home environments, combining high-sensitivity sensing with energy-autonomous data transmission.

To bridge the gap between flexible sensing and high-precision motion capture, Wu et al. developed an insole-shaped pressure sensor array using a fabric-based piezoresistive mechanism [117]. The sensors feature an exceptional sensing range of 3770.9 kPa and a high sensitivity of 2.68 kPa^−1^, supported by an ultra-fast response time and a cycle life exceeding 4 million repetitions, ensuring long-term stability across diverse mechanical loads. By deploying a 1D-CNN-based deep learning framework for posture classification and a transformer-based architecture for pose estimation, the system achieves a 95.5% classification accuracy for 10 distinct postures and a joint position prediction accuracy of ~3.6 cm. The system operates at a sampling rate of ~30 Hz, utilizing 1 s time windows for posture classification and 2 s windows for pose estimation. While the model for general posture classification is pre-trained and robust for diverse users, the high-precision pose estimation task requires a brief per-user fine-tuning session. Ultimately, this synergy between ultra-wide-range fabric sensors and specialized AI architectures provides a high-fidelity, wearable solution for continuous and unobtrusive lower limb motion capture in complex real-world environments.

In summary, the synergistic optimization of sampling rate, window length, and overlap ratio constitutes a critical factor in achieving a balanced trade-off among performance metrics. While increasing the sampling rate, window length and overlap rate generally enhances recognition accuracy by providing higher signal fidelity and richer temporal features, it inherently increases computational load and data-gathering latency. The window length should be aligned with the intrinsic periodicity of the target activity. Furthermore, the recommended window overlap rate typically ranges from 50% to 75% [79,116]. The selection of these parameters must be task-specific, balancing the need for high-precision diagnostic data against the stringent low-latency requirements of real-time human–machine interaction.

From a broader perspective, the distinction between universal and personalized tasks in flexible wearable motion and posture sensing arises fundamentally from biomechanical heterogeneity. High-level activities, including walking, running, and basic posture transitions such as sitting to standing, tend to exhibit population-consistent signal patterns and can therefore often be addressed using pretrained generalized models with minimal or no retraining. In contrast, tasks involving fine-grained motion decoding, including subtle gesture recognition, absolute joint-angle estimation, and precise gait analysis, are inherently user dependent, owing to compounded effects of sensor placement variability, individual anatomy, and subject-specific skin–electrode coupling in flexible interfaces. Collectively, these observations underscore that while coarse activity recognition may be achieved without explicit retraining, extracting reliable, high-fidelity biomechanical information from flexible wearables typically necessitates retraining or personalization-aware adaptation [79,118,119].

## 5. Conclusions and Perspectives

AI technology has fundamentally reshaped the development paradigm of wearable flexible sensors, enabling intelligent, multimodal systems for continuous human motion and posture monitoring [118,120]. This paper reviews and discusses the application of AI-enhanced wearable sensors in human motion monitoring. We first outlined representative human motion recognition approaches and advances in flexible sensor technologies, followed by a discussion of AI-driven methods for extracting meaningful information from complex sensor data. Subsequently, we discussed the distinct characteristics of different body parts and highlight representative applications of flexible sensors for both large-range limb movements and subtle physiological motions, such as eye blinking and throat vibration. Collectively, these studies demonstrate the versatility and critical role of flexible wearable sensors in next-generation human motion monitoring systems. Looking ahead, wearable flexible sensing systems are expected to evolve beyond isolated sensing elements toward fully integrated, intelligent platforms capable of perception, learning, and adaptive response. This evolution will be driven by coordinated advances in sensor materials and structures, energy management strategies, artificial intelligence algorithms, and system-level integration.

At the device level, the performance, robustness, and application scope of flexible sensors are fundamentally governed by material selection and structural design. Continued progress will rely on the development of advanced soft functional materials and composites to enhance sensitivity, response speed, mechanical durability, and long-term stability [121,122,123]. However, standardized evaluation frameworks for long-term stability and measurement accuracy remain underdeveloped for flexible wearable sensors. Unlike conventional industrial sensors, widely accepted accuracy classifications have not yet been established, and most studies report only short- or mid-term drift under controlled laboratory conditions. The development of unified benchmarking protocols for hysteresis, temperature effects, and long-term stability will be essential to support reliable real-world deployment. For deployment in outdoor, industrial, or extreme environments, sensors must maintain reliable performance under temperature fluctuations, humidity, ultraviolet radiation, and chemical exposure, motivating the development of protective coatings and environmentally resilient substrates. In medical and healthcare scenarios, biocompatibility and biosafety are critical considerations [122]. Future systems may incorporate biodegradable or bioresorbable materials, enabling transient or self-degradable sensors that minimize long-term physiological burden when implanted. Beyond sensing, flexible devices are expected to integrate self-calibration, self-diagnosis, and self-adaptation capabilities through embedded microprocessors, wireless communication modules, and on-device intelligence. As wearable sensors increasingly handle sensitive health and behavioral data, ensuring data security and system reliability will be essential. Emerging solutions such as blockchain-based architectures have shown promise for secure data storage and transmission in medical-grade wearable systems, helping to protect user privacy and data integrity [124].

Energy autonomy represents another fundamental challenge for long-term wearable motion sensing. Self-powered sensors that harvest energy from the surrounding environment offer a viable pathway toward sustainable and maintenance-free operation. Future research could focus on flexible energy storage and harvesting technologies, including stretchable batteries, supercapacitors, and advanced energy-harvesting materials and devices. By integrating energy-harvesting components directly into sensor architectures, wearable systems can generate power from ambient sources such as body motion, thermal gradients, and light [125]. Hybrid energy-harvesting strategies that combine multiple mechanisms are expected to improve power stability under variable operating conditions. Complementing hardware advances, intelligent energy management will play a crucial role in optimizing energy generation, storage, and consumption. Machine learning-based energy management algorithms can enable adaptive control of system operation, dynamically balancing sensing performance and power consumption to maximize energy utilization efficiency [126].

Beyond individual sensors or single sensing modality, future intelligent wearable motion sensing systems will increasingly rely on multimodal integration. Human motion and posture are inherently complex phenomena involving coordinated mechanical deformation, muscle activation, inertial dynamics, and often physiological responses. The integration of heterogeneous sensing modalities can provide a more comprehensive and robust description of human kinematics. However, multimodal sensing also introduces substantial challenges, including signal synchronization, cross-modal interference suppression, feature alignment, and data fusion. Addressing these issues will require advances in signal preprocessing, representation learning, and algorithm optimization, with AI playing a central role in extracting complementary information across modalities while maintaining robustness in real-world conditions. As the number of sensors and sensing modalities increases, system-level challenges related to communication, synchronization, and data collection across distributed body locations become increasingly prominent. In this regard, the emerging concept of metamaterial or smart clothing offers a promising pathway toward scalable body sensor networks for simultaneous multi-point motion tracking [127,128]. By embedding conductive threads or functional textiles into garments, wireless communication networks operating at designated frequencies can be established, enabling seamless integration of sensing, communication, and wearability.

At the system intelligence level, most current wearable sensing platforms rely heavily on cloud-based processing, which enables complex model execution but introduces latency, privacy risks, and dependence on continuous connectivity. As edge computing technologies mature, future wearable systems are expected to shift toward localized AI processing. By embedding lightweight AI models and dedicated inference hardware at the sensor or device level, wearable systems can achieve real-time responses for motion recognition and anomaly detection while reducing communication overhead and power consumption. Edge-based processing further enables sensitive biometric data to be analyzed locally, transmitting only essential information and thereby significantly mitigating privacy risks. This transition will require the development of compact and energy-efficient AI models through techniques such as model compression and knowledge distillation. Lastly, human motion patterns exhibit substantial inter-individual variability and evolve over time due to fatigue, aging, injury, or rehabilitation. Future wearable systems should therefore move beyond static, population-level models toward adaptive and personalized learning frameworks. Continual learning, self-supervised learning, and calibration-free or minimally supervised approaches are expected to play a pivotal role in maintaining long-term performance while minimizing the burden of data labeling and user intervention.

In summary, the future of AI-enhanced flexible sensors for human motion and posture sensing lies in the deep integration of multimodal hardware, intelligent algorithms, and efficient deployment strategies. Continued interdisciplinary efforts spanning materials science, biomechanics, signal processing, and artificial intelligence will be essential to realize wearable systems that are not only accurate and robust, but also adaptive, interpretable, and scalable for real-world use.

## Figures and Tables

**Table 1:** Applications of AI in Flexible Sensors.

Ref.	Sensing Mechanism	Sensitivity	Sensing Range	ML Model Type	Model Function	Applications
[79]	Inertial/EMG	N.A.	±3 g, ±400°/s	FCN, CNN, RNN	Denoising	Healthcare, Robotics, Human–Machine Interfaces
[80]	Piezoresistive	GF = 243.6	0–77%(strain)	CWT-CNN	Classification	Tire pressure detection, Speech recognition
[81]	Capacitive	1.64 kPa^−1^	0–60 kPa	1D-CNN	Classification	Facial expression recognition
[82]	Piezoresistive	GF = 126.42, 24.21 mV/kPa	0–183% (strain), 0–208 kPa	CNN-LSTM	Classification	Aphasic intelligent interaction
[83]	Triboelectric	N.A.	20–50 kPa	1D-CNN	Classification	Robotic tactile sensing
[84]	Piezoelectric/Triboelectric	34 mV/kPa	0–170 kPa	2D-CNN	Classification	Human gait analysis, Activity monitoring
[85]	Piezoresistive	1.46 × 10^6^ kPa^−1^	0–89 kPa	ANN	Classification	Virtual Reality, Prosthetics, Sports Science, HMI
[86]	Piezoresistive	1.16 kPa^−1^	0–1.5 MPa	CNN	Classification	Health Monitoring, Human–Machine Interface
[87]	Piezoresistive	356 kPa^−1^ (0–2 kPa)	0–3.3 MPa	MK-ResCNN	Classification	HMI, Patient-audience dialogue system, Health monitoring
[88]	Piezoresistive	7.44 kPa^−1^ (2–20 kPa)	0–240 kPa	ResNet-50	Classification	Smart Insoles, Gait Analysis, Posture Recognition
[89]	Piezoresistive	3656.8 kPa^−1^ (0–100 kPa)	0–3 MPa	1D CNN-BiLSTM-Attention	Regression	Health Monitoring, Sports Performance Evaluation
[90]	Piezoresistive	0.046 kPa^−1^ (0.03–15 kPa)	0–100 kPa	CNN	Classification	Healthcare Monitoring, Rehabilitation Engineering

EMG, electromyography; N.A. means this data was not mentioned in the literature; FCN, fully convolutional network; CNN, convolutional neural network; RNN, recurrent neural network; CWT, continuous wavelet transform; LSTM, long short-term memory; ANN, artificial neural network; MK-ResCNN, multi-kernel residual convolutional neural network; BiLSTM, bidirectional long short-term memory; 1D, one-dimensional.

## Data Availability

No new data were created or analyzed in this study.
